# Retraction of: Extracellular vesicle derived miR-544 downregulates expression of tumor suppressor promyelocytic leukemia zinc finger resulting in increased peritoneal metastasis in gastric cancer

**DOI:** 10.18632/aging.206324

**Published:** 2025-10-31

**Authors:** Wencheng Kong, Xinchun Liu, Guang Yin, Sixin Zheng, Akao Zhu, Panpan Yu, Yuqiang Shan, Rongchao Ying, Jian Zhang

**Affiliations:** 1 Department of General Surgery, Affiliated Hangzhou First People’s Hospital, Zhejiang University School of Medicine, Hangzhou 310006, Zhejiang Province, P.R. China

**Keywords:** peritoneal metastasis, gastric cancer, PLZF, miR-544, extracellular vesicle

**This article has been retracted:** Aging has completed its investigation of this paper. Several concerns were raised about the paper, including duplications and overlaps with unrelated papers from different institutions, as well as the reuse of previously published data. Specifically, the GAPDH Western blot image in Figure 1B is identical to the b-actin image in Figure 3D of an unrelated, earlier published paper [1]. Similarly, the PLZF blot in Figure 6B matches the USF1 blot in Figure 5A of the same paper [1]. Furthermore, immunofluorescence images from Figure 3D were previously published in Figure 5K of [2] and Figure 5B of [3]. Article [3] also shares a transwell assay image in Figure 5B with a panel in Figure 3D. Additionally, transwell assay images from Figure 3 appear in earlier published papers [4–12]. The transwell assay images from Figure 7 duplicate those found in articles [13–15], all of which have already been retracted. We also identified three later published articles that utilized some of the transwell images from Figures 3 and 7 [16, 17], and immunofluorescent images from Figure 3D [18]. The Scientific Integrity office at Aging contacted the authors regarding these issues. Dr. Wencheng Kong, the first author, requested a correction but did not follow up. In light of these findings, the Editors decided to retract the paper. The authors were informed of this decision and all authors agreed to retract the manuscript.
